# Response: Rectovaginal septum is a real location for deep endometriosis; comment on the article: ‘the myth of rectovaginal septum endometriosis’

**DOI:** 10.1530/RAF-26-0116

**Published:** 2026-07-15

**Authors:** Mathew Leonardi, Shay M Freger

**Affiliations:** McMaster University – Department of Obstetrics and Gynecology, Hamilton, Ontario, Canada

**Keywords:** rectovaginal septum, endometriosis, ultrasound, laparoscopy

Dear Editor,

We read Prof. Szabó’s response to our ‘Research Letter’ with interest and curiosity. The summation of the response is that rectovaginal septum (RVS) endometriosis is common, but isolated RVS endometriosis is rare (Szabó 2026). This directly contradicts the results of the published Research Letter, which claims that true RVS endometriosis is either very rare or possibly non-existent (Leonardi *et al.* 2026).

While we agree that there is a certain logic to infer that inferior infiltration into the RVS occurs, this is simply not corroborated by our data (Mick *et al.* 2025). At most, deep endometriosis (DE) lesions of the very caudal aspect of the peritoneal reflection could be considered as ‘infiltrating’ the very cephalad aspect of the RVS. However, when these exist in the absence of otherwise severe DE and adhesions, the actual RVS is relatively simple and straightforward to open, as a potential space, revealing the nature of these lesions as ‘peritoneal’, and specifically, we would label these as rectouterine pouch peritoneal lesions. Even in cases of severe posterior compartment adhesions (e.g. obliteration), surgically reaching and opening the RVS is generally thought of, among advanced gynecologic surgeons, as a ‘moment of freedom’, below the level of the severe lesions and adhesions. We consider the more accurate, less conflated localization of these lesions, which may be at the cephalad aspect of the RVS, to be ‘rectouterine pouch peritoneum’.

Unfortunately, referencing data based on the IDEA consensus statement requires caution, as the original IDEA consensus statement clearly did not fully appreciate the difference between the RVS and rectouterine pouch peritoneum (or the lateral uterosacral ligament (USL)), as exhibited by their Fig. 5, which includes at least two (of three) images characterizing RVS lesions that are clearly intraperitoneal, as evidenced by the adjacency and relative location of the free fluid in the peritoneal cavity (Guerriero *et al.* 2016). Moreover, there is a possibility that the dotted red and blue lines depicted in the original IDEA consensus statement Fig. 4, which aim to delineate anatomical spaces, including the RVS, require reconsideration, seeing as the area between red and blue dotted lines is also RVS (in the opinion of the authors) but labeled as posterior vaginal fornix. As such, it is reasonable to consider that contributors to the IDEA database and subsequent studies may be falsely labeling lesions of the USL or rectouterine pouch peritoneum as RVS.

These authors appreciate that this concept, originally published in the Research Letter, may seem unlikely given traditional teachings, published rates of publication, and global nomenclature. Our intention is to encourage critical thinking of old dogmas, separate the overlapping and conflicting terms ‘RVS’ and rectovaginal endometriosis, and draw attention to advancements in our knowledge of anatomy since the 2016 IDEA consensus statement. In the well-established and increasingly advanced era of imaging and surgery as complementary methods for ‘viewing’ endometriosis and the corresponding anatomy, it should not be surprising but rather expected that our understanding of this enigmatic disease will dramatically evolve.

## Declaration of interest

The authors declare that there is no conflict of interest that could be perceived as prejudicing the impartiality of the work reported.

## Funding

This research did not receive any specific grant from any funding agency in the public, commercial, or not-for-profit sector.
